# Genomic Features and Insights into the Taxonomy, Virulence, and Benevolence of Plant-Associated *Burkholderia* Species

**DOI:** 10.3390/ijms20010121

**Published:** 2018-12-29

**Authors:** Mohamed Mannaa, Inmyoung Park, Young-Su Seo

**Affiliations:** 1 Department of Integrated Biological Science, Pusan National University, Busan 46241, Korea; mannaa_mohamed@yahoo.com; 2 Department of Oriental Food and Culinary Arts, Youngsan University, Busan 48015, Korea; inmpark@ysu.ac.kr

**Keywords:** *Burkholderia* genomics, phytopathogenic *Burkholderia*, plant symbiotic *Burkholderia*

## Abstract

The members of the *Burkholderia* genus are characterized by high versatility and adaptability to various ecological niches. With the availability of the genome sequences of numerous species of *Burkholderia*, many studies have been conducted to elucidate the unique features of this exceptional group of bacteria. Genomic and metabolic plasticity are common among *Burkholderia* species, as evidenced by their relatively large multi-replicon genomes that are rich in insertion sequences and genomic islands and contain a high proportion of coding regions. Such unique features could explain their adaptability to various habitats and their versatile lifestyles, which are reflected in a multiplicity of species including free-living rhizospheric bacteria, plant endosymbionts, legume nodulators, and plant pathogens. The phytopathogenic *Burkholderia* group encompasses several pathogens representing threats to important agriculture crops such as rice. Contrarily, plant-beneficial *Burkholderia* have also been reported, which have symbiotic and growth-promoting roles. In this review, the taxonomy of *Burkholderia* is discussed emphasizing the recent updates and the contributions of genomic studies to precise taxonomic positioning. Moreover, genomic and functional studies on *Burkholderia* are reviewed and insights are provided into the mechanisms underlying the virulence and benevolence of phytopathogenic and plant-beneficial *Burkholderia*, respectively, on the basis of cutting-edge knowledge.

## 1. Introduction

The genus *Burkholderia* belongs to the subphylum of β-proteobacteria and encompasses Gram-negative bacterial species with high genetic versatility and adaptability to various ecological niches [1]. More specifically, they are able to occupy ecosystems as diverse as soils, plants, and even animal and human bodies as serious disease-causing agents [2,3,4]. Recently, *Burkholderia* organisms have drawn increasing attention as abundant and fundamental components of various ecosystems. The first member of the genus was isolated in 1942 from carnations displaying wilt and root-rot symptoms. It was initially named *Phytomonas caryophylli* and later renamed *Pseudomonas caryophylli* [5]. In 1950, another species was isolated from onions showing sour skin rot and named *Pseudomonas cepacia* [6,7]. Some members, originally included in the genus *Pseudomonas*, were later reclassified in the new *Burkholderia* genus due to improved taxonomic tools, the availability of techniques for DNA–DNA hybridization, as well as for 16S rRNA analysis [8]. Since then, many other species have been, and are still being, described and added to the genus.

The adaptability of *Burkholderia* spp. to diverse environments and their ecological versatility rely on the high genetic plasticity of their multi-chromosome genomes, rich in insertion sequences [9] and exhibiting a relatively high proportion of coding regions, allowing these bacteria to produce various metabolites with degradative potential [10]. Such metabolic capacity is a fundamental feature for their survival and fitness in different habitats [11].

The *Burkholderia cepacia* complex (BCC), one of the major components of the *Burkholderia* genus, comprises a group of related opportunistic human pathogen species responsible for pulmonary infections, particularly in immunocompromised and cystic fibrosis patients [9]. Due to the importance of BCC members as human pathogens, intensive research efforts and discussion have been devoted to the epidemiology of human diseases caused by *Burkholderia* spp. Other related species include animal pathogens such as *Burkholderia mallei* and plant pathogens such as *Burkholderia glumae*, *Burkholderia gladioli*, and *Burkholderia plantarii*. Notably, another group of plant-associated *Burkholderia* spp. comprises potentially beneficial species of plant growth-promoting or plant-symbiotic bacteria, favoring the healthy growth of plants by nitrogen fixation and protection against plant diseases. From the taxonomic standpoint, an effort is being made to better discriminate beneficial from pathogenic *Burkholderia* spp. [12].

In this review, the taxonomy of the *Burkholderia* genus is discussed, highlighting the major updates and the proposals of new delineated genera. Moreover, studies on the unique features of *Burkholderia* genomes, related to bacterial adaptability to a wide-range of ecological niches, are reviewed. The main focus of this review is on plant-associated species of the *Burkholderia* genus, including both phytopathogenic and beneficial symbiotic species, with a special attention to factors affecting virulence and benevolence, respectively. However, the insect-symbiotic *Burkholderia* is not covered in this review.

## 2. Taxonomic Updates of the *Burkholderia* Sensu Lato

Following the proposal of *Burkholderia* as a new genus in 1992 [8], and the continuous increase in the number of species within it, several taxonomic rearrangements have been suggested. Pieces of evidence from 16S rRNA analysis and the construction of phylogenetic trees based on other genes, such as *recA*, have increasingly supported the division of the *Burkholderia* genus into two distinct lineages [13]. Based on the suggested division, one group comprises human, animal, and plant pathogens or opportunistic pathogens, whereas the other group is represented by environmental xenobiotic-biodegrading, plant symbiotic, and plant growth-promoting species. 

The distinction between the two lineages has been further supported by the identification of genomic within-group similarities and between-group differences. Ussery et al. [14] explored the genomic diversity among members of the *Burkholderia* genus and defined the pan- and core-genomes using the genome sequences available at the time of that study (56 full and partial genome sequences). A phylogenetic analysis conducted on 612 genes from the core *Burkholderia* genome resulted in a clear separation of the pathogenic *Burkholderia* spp. from members of the other clade. By combining comparative genomics with phylogenetic analyses based on 16s rRNA, *recA*, *gyrB*, and *acdS* genes, Gyaneshwar et al. [15] suggested the distinction of the *Burkholderia* genus into two subgenera and initially proposed the new genus, *Caballeronia*.

Consistently, multilocus sequence analysis using four housekeeping genes, namely *atpD*, *gltB*, *lepA*, and *recA*, in combination with 16S rRNA, confirmed the presence of different *Burkholderia* lineages by separating the genus into two major clusters [16]. The distinction was even more obvious after bioinformatics analysis of the virulence loci of *Burkholderia* genomes and functional pathogenicity tests [17]. In light of compelling evidence on the existence of systematic differences within the *Burkholderia* group, a novel genus, *Paraburkholderia*, was proposed based on the analysis of conserved sequence insertions/deletions [18]. Since then, several other species have been ascribed to the *Paraburkholderia* genus and the previously proposed *Caballeronia* genus [19]. Following reassessment of *Burkholderia andropogonis*, this species was separated into a new genus, *Robbsia*, as confirmed by phylogenetic and comparative genomic analysis [20]. Using whole-genome sequence data, Beukes et al. [21] addressed these complex phylogenetic relationships and confirmed the existence of generic boundaries among *Burkholderia* sensu lato bacteria. Although the analyzed genomes were similar in size and number of encoded genes, the genomes of *Burkholderia* sensu stricto showed higher G+C contents. The latter study includes a large-scale phylogenetic analysis on 106 common genes of *Burkholderia* sensu lato, specifically selected to limit phylogenetically irrelevant data. The results support the subdivision of *Burkholderia* sensu lato into five distinct lineages: *Burkholderia* sensu stricto, *Paraburkholderia*, *Caballeronia*, *Robbsia*, and *Paraburkholderia rhizoxinica* [21]. More recently, whole-genome analysis has been employed in the context of an international joint effort to investigate the taxonomic status of selected species of *Burkholderia* sensu lato [22]. Based on the phylogenetic analysis of conserved genes and average nucleotide and amino acid identities, two novel genera were proposed within the *Burkholderia* sensu lato assemblage. The first, *Mycetohabitans*, encompasses fungal symbiotic species with relatively small genomes. The second, *Trinickia*, includes soil and plant-associated species. Furthermore, the comparison of core-genomes revealed the presence of functional genus-specific genes. A historical overview of the major rearrangements and updates in *Burkholderia* taxonomy is summarized in Table 1.

**Figure 1:**
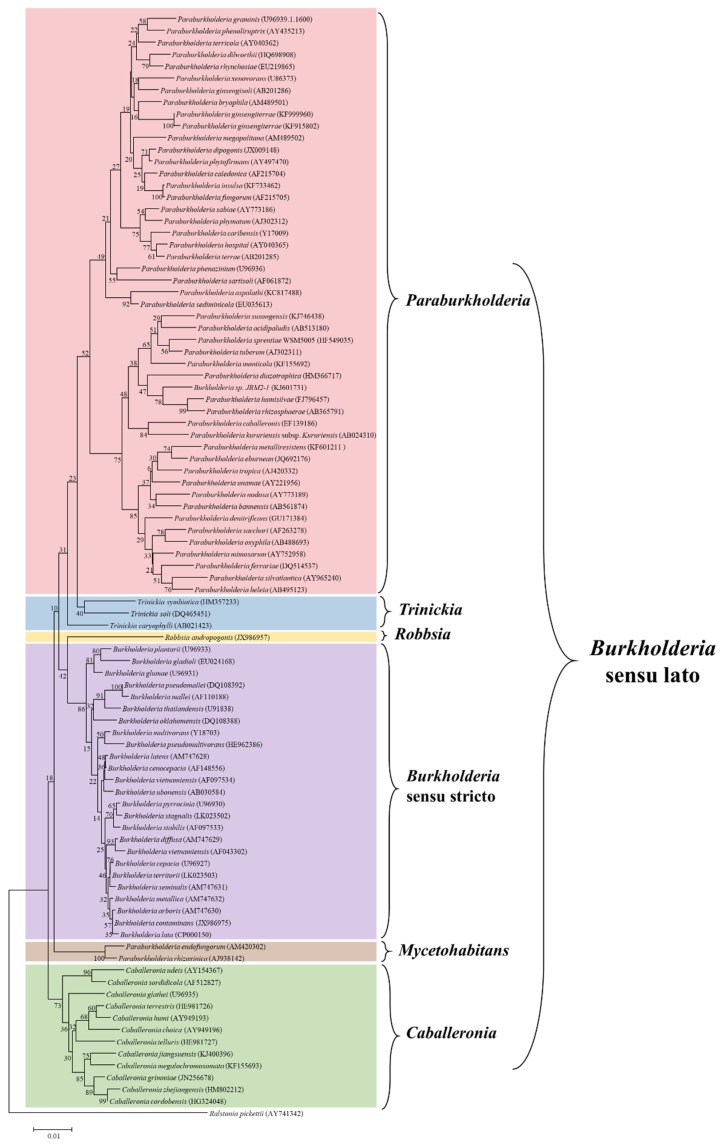
Phylogenetic tree constructed by the neighbor-joining method using 16S rRNA sequences of representative strains of *Burkholderia* sensu lato assemblage. The 16S rRNA sequences were retrieved from the SILVA database (https://www.arb-silva.de/). Species names were corrected to match the current taxonomic positions and the accession numbers for each sequence are given in parenthesis following the species name. The updated genera and their phylogenetic relationships are also shown. *Ralstonia pickettii* (AY741342) was used as an outgroup.

It is clear that the *Burkholderia* sensu lato group has undergone several taxonomic changes and could be subjected to further adjustments in the future. The increased availability of genomic information, as well as the advancements in bioinformatics analysis, have enhanced our understanding of the evolutionary and taxonomic relationships between clinically, environmentally, industrially, and agriculturally important *Burkholderia* species. All this considered, the large group of *Burkholderia* sensu lato is currently composed of six genera, i.e., *Burkholderia* sensu stricto, *Caballeronia*, *Paraburkholderia*, *Robbsia*, *Mycetohabitans*, and *Trinickia*. The six genera were established after phylogenetic analysis of the main representative *Burkholderia* sensu lato strains using the neighbor-joining method, based on 16S rRNA sequences retrieved from the SILVA database (https://www.arb-silva.de/), as shown in Figure 1. 

With regard to the scope of the current review, phytopathogenic *Burkholderia* species are classified in the *Burkholderia* sensu stricto group, closely related to the *Burkholderia cepacia* complex; the former *Burkholderia andropogonis* is currently a separate genus as *Robbsia andropogonis*; and *Burkholderia caryophylli* is now classified as *Trinickia caryophylli*. On the other hand, most of the plant-beneficial *Burkholderia* species were transferred to the *Paraburkholderia* and *Caballeronia* genera.

## 3. Genomic Features of the Plant-Associated Pathogenic and Beneficial *Burkholderia*

Because of the clinical importance of the *B. cepacia* complex, special consideration is devoted to these bacteria by genomic studies and, therefore, their genomes are well-characterized [9]. Besides common genomic features shared by most organisms of the large *Burkholderia* sensu lato group, functional features specific to the plant-associated groups are present and allow them to occupy various ecological niches with different lifestyles, as pathogens, legume nodulators, or biocontrol agents. Common features of *Burkholderia* species include relatively large multi-replicon genomes, the presence of genomic islands, and multiple insertion sequences conferring high genome plasticity [9,10].

### 3.1. Genome Size

Generally, *Burkholderia* organisms have larger genomes (7–8 Mbp on average) than other bacteria; however, the genome size greatly varies among different *Burkholderia* groups. The smallest genome among the free living *Burkholderia* (3.75 Mbp) is found in the fungal endosymbiont, *Mycetohabitans rhizoxinica* (basonym *Burkholderia rhizoxinica*), whereas the largest genome (11.5 Mbp) is found in the soil bacterium *Paraburkholderia terrae* (basonym *Burkholderia terrae*). It is suggested that genome size can provide insights into the evolutionary history and the relationships between *Burkholderia* species. It is also noted that the particular lifestyle of *Burkholderia* species might reflect selective pressure for a specific genome size [23]. 

Two main models for genome evolution in *Burkholderia* species were proposed: (i) plasmids might undergo rearrangements following parallel transfer, becoming established as additional chromosomes or megaplasmids and allowing for bacterial acquisition of essential genes; and (ii) loss of genomic information following rearrangements [24]. An example of the latter mechanism could be *M. rhizoxinica*, the obligate endosymbiont of the zygomycete *Rhizopus microsporus*, which is a causal agent of rice seedling blight development by production of rhizoxin [25]. The genome of *M. rhizoxinica*, containing one chromosome, a megaplasmid, and a plasmid, is remarkably small (relative to other *Burkholderia*), most likely because of rearrangements and deletion of genomic information, especially in genes coding for mobile genetic elements. The endosymbiotic lifestyle reduced the bacterial capacity to adapt to varying habitats and made efficient sensing superfluous [26,27]. On the other hand, a large part of the chromosomal coding region is devoted to the production of the secondary metabolite, rhizoxin [26,27]. 

Regarding the plant symbiotic *Burkholderia*, which can also be free-living in the bulk soil or water, a characteristic large genome size could explain their versatile lifestyle and the capacity to perform many plant-beneficial functions [28].

In another comparative genomic study of phytopathogenic *Burkholderia* species, Seo et al. [29] analyzed the genome sequences of different strains of *B. glumae*, *B. gladioli*, and *B. plantarii* and found genome size variations not only between species but also between different strains of the same species. For *B. glumae*, the smallest genome size, ~4.9 Mbp, was exhibited by the AU6208 strain, whereas the largest, ~7.2 Mbp, was found in the BGR1 strain. The relatively small genome size of *B. glumae* AU6208 could be attributed to genome rearrangements or deletions, as a result of adaptation to different hosts. Notably, although pathogenic to rice, AU6208 was originally isolated from infant patients, unlike other strains that were all isolated from rice as the original host. The genome size of *B. gladioli* BSR3 is ~9 Mbp and that of *B. plantarii* is ~8 Mbp. 

### 3.2. Multi-Replicon Nature

A multi-replicon genome structure is found in most *Burkholderia* species with various numbers of chromosomes and plasmids. Martínez-Aguilar et al. [28] studied the multi-replicon genomes of plant-associated diazotrophic *Burkholderia* species and found that three strains of *Paraburkholderia unamae* and *Paraburkholderia silvatlantica* (formerly *Burkholderia* spp.) contain genomes of four replicons. The largest and smallest replicon sizes are ~3.31 and ~1.11 Mbp, respectively. In addition, the genomes of three strains of *Paraburkholderia tropica* (formerly *Burkholderia tropica*) contain five replicons ranging from ~3.24 to ~0.53 Mbp. The authors suggested that such multi-replicon large genomes could explain the capacity of these diazotrophic *Burkholderia* species to efficiently colonize the rhizosphere as well as endophytic environments.

A multi-replicon genome structure is also reported among the plant-pathogenic *Burkholderia* species. The genomes of the representative strains *B. glumae* BGR1 and *B. gladioli* BSR3 contain two chromosomes and four plasmids, while the *B. plantarii* ATCC43733 genome contain two chromosomes and one plasmid [29]. Agnoli et al. [30] found that deletion of the third chromosome from the genomes of several BCC species results in the attenuation of virulence and the loss of many functions such as the ability to utilize several substrates, antifungal activity, and biosynthesis of extracellular polymeric substances. Intriguingly, bacterial viability is not significantly affected, despite the massive loss of genomic information (~1 Mbp). This finding indicates that multi-replicon genomes are essential for adaptation but not necessarily for bacterial growth and survival.

While studying the pan- and core-genomes of *Burkholderia* species, Ussery et al. [14] found that only few hundred genes are conserved among all genomes to form the *Burkholderia* core genome, whereas more than 40,000 gene families are present in the *Burkholderia* pan genome. A more recent study of the available genome sequences, focusing on 110 genome sequences, shows that around 35,680 genes belong to pan-gene and 717 to core-gene families [23]. In the latter study, the number of shared genes has significantly dropped upon adding genes from different replicons to the analysis, which indicates the low percentage of genes shared by different genomic replicons of *Burkholderia*. In the same context, Seo et al. [29] analyzed the genomes of 106 *Burkholderia* species, representing the different groups, and found that 78,782 genes correspond to pan-genomes, while 587 are core genes conserved in all tested species. By considering the genomes of several strains of three plant pathogenic *Burkholderia* (*B. glumae*, *B. gladioli* and *B. plantarii*), 12,758 pan-genome genes are present in the tested strains, while 1908 genes are found conserved as the core-genome [29]. 

### 3.3. Genomic Islands and Multiple Insertion Sequences

Along with the above-mentioned features of *Burkholderia* genomes, other unique features are related to the genetic plasticity of these bacteria, such as the presence of genomic islands and multiple insertion sequences. Genomic islands refer to foreign DNA regions that have been horizontally acquired and became integrated within the genomes of several *Burkholderia* species. They have important evolutionary roles and can specify several accessory functions [31]. Multiple approaches have been used to identify these foreign DNAs, such as the analysis of G+C content, which has been found to differ in genomic islands with respect to the rest of the genome [32,33]. Genomic islands vary in their structure and content between *Burkholderia* species and account for more than 10% of the genomes in most species [24]. Genomic islands linked to pathogenicity and transmission have been extensively characterized. However, the coding capacity of genomic islands is not only associated with pathogenicity and virulence but also with adaptive traits such as symbiosis and metabolism [31]. An example of this is the environmental species *Paraburkholderia xenovorans* (formerly *Burkholderia xenovorans*), in which more than 20% of total genes are a recent acquisition and mainly involved in niche adaptation [34]. 

The genomes of *Burkholderia* species also contain many diverse insertion sequences that are mainly attributed to genomic rearrangements and replicon fusion and are related to transcriptional regulation [10,24,35,36]. This finding indicates that the presence of multiple insertion sequences in *Burkholderia* genomes has a role in phenotypic, as well as genetic diversity, by activating and/or inactivating adjacent genetic loci. Target genes may be inactivated or, in the case of adjacent promoter-less genes, activated by the promoters provided by insertion sequences [37]. Furthermore, the presence of multiple copies of the same insertion sequence may drive genetic rearrangements by fusion of two replicons, inversion, deletion, and duplication of the intervening region [38,39]. Accordingly, the transposition of insertion sequences is a very important evolutionary mechanism for the emergence of adaptation capacity and variability in *Burkholderia* species. A clear example of insertion sequence-mediated genomic alterations is provided by the genome of *B. mallei*, which is suggested to arise from several deletions and inversions of the *Burkholderia pseudomallei* genome. Evidence supporting such an assumption includes the presence of insertion sequences bordering most of the breakdown points and the presence of regions from chromosome 1 of *B. pseudomallei* on chromosome 2 of *B. mallei* [40]. In the phytopathogen *B. glumae*, the insertion sequences IS1417, IS1418, and IS1419 are characterized and found to be prevalent among different field strains. Apparently, these sequences may have been horizontally acquired by *B. glumae* from other related species, as they are also found in species such as *B. gladioli* and *B. plantarii*, with IS1417 being the most prevalent [41].

## 4. Plant Pathogenic *Burkholderia*

The *Burkholderia* group comprises several etiological agents of plant diseases causing significant economic losses by affecting the production of various crops. The major phytopathogenic species of *Burkholderia* group are *B. glumae*, *B. gladioli*, *B. plantarii*, *R. andropogonis*, and *Trinickia caryophylli* (formerly *Burkholderia caryophylli*) [42]. Although the mechanisms involved in pathogenicity and adaptation have not been fully elucidated, genomic studies and functional analyses, accompanied by phenotypic characterization, have enhanced our understanding of the virulence factors involved [29]. 

*B. glumae*, causing bacterial panicle blight and seedling rot of rice, was first reported in Japan, and is currently considered a major threat to rice cultivation worldwide [43,44]. In rice, the seed borne nature of *B. glumae* facilitates disease spread, which is estimated to cause the loss of up to 75% of heavily infested fields [45]. *B. glumae* is also found to be responsible for wilting symptoms in other hosts including pepper, sunflower, and tomato [46]. 

*B. gladioli* was first reported to cause corm rot in *Gladiolus* spp. [47]. Later, three pathovars were found: *B. gladioli* pv. *gladioli*, causing gladiolus rot; *B. gladioli* pv. *alliicola*, causing onion bulb rot; and *B. gladioli* pv. *agaricicola*, causing rapid soft rot of cultivated mushrooms [48,49,50]. 

*B. plantarii* was first reported to cause rice seedling blight in Japan and has been isolated from several other host plants [42,51]. Both *B. plantarii* and *B. gladioli* have also been reported to cause, in rice, a similar disease to that caused by the related species *B. glumae* [51,52]. 

*R. andropogonis* (formerly known as *B. andropogonis*) was first reported to be the causative agent of strip disease in sorghum and leaf spot in velvet bean, and since then it has been described to cause disease in a wide range of host plants [42,53,54,55]. 

*T. caryophylli* (formerly *B. caryophylli*) is responsible for a damaging disease of carnation in Japan, causing significant losses and was also found to cause onion rot [56,57]. 

The well-characterized fungal endosymbiont *M. rhizoxinica* (formerly *B. rhizoxinica*) could also be considered as a phytopathogen due to its cooperation with the phytopathogenic fungus *Rhizopus microsporus* in the development of rice seedling blight [42]. *M. rhizoxinica* controls the vegetative growth of the fungus [58]. However, although the main virulence factor of rice seedling blight was originally thought to be produced by the fungus, it was later revealed that *M. rhizoxinica* is responsible for rhizoxin production [59]. 

### 4.1. Virulence Factors in Phytopathogenic Burkholderia

The first isolated members of *Burkholderia* group were phytopathogens with unique genomic features, as described above, enabling them to develop an arsenal of virulence factors, thereby causing diseases in various host plants [29,42]. The pathogenicity of phytopathogenic members of the *Burkholderia* group is complex and several virulence factors have been found to contribute to disease development. The virulence factors and their genetic regulation in *B. glumae* have been extensively studied due to the significant economic impact of bacterial panicle blight and bacterial grain rot on rice cultivations worldwide [44,60]. The major virulence factors include phytotoxins and other virulence-related enzymes, bacterial motility, secretion systems, and exopolysaccharides (EPSs). Some of these virulence factors are common among phytopathogenic *Burkholderia*, whereas others are species-specific. 

#### 4.1.1. Phytotoxins

Phytotoxins are plant-deleterious compounds produced by pathogens and one of the major responsible factors for the development of disease symptoms [61]. Toxoflavin, the most important and studied phytotoxin of phytopathogenic *Burkholderia* species, was first described as an azapteridine antibiotic in *Burkholderia cocovenenans* [62], later found in *B. glumae* [63], and more recently in the related species *B. gladioli* [64]. Toxoflavin from *B. glumae* is associated with the inhibition of sprout and root elongation in rice seedlings, as well as severe damage in toxin-treated panicles [65]. Moreover, Jeong et al. [46] showed that toxoflavin is also involved in development of severe wilting symptoms in several other crops, indicating that a broad range of host plants are affected. Toxoflavin is also toxic to mice and exhibits antibacterial and antifungal activities [66]. The wide spectrum of toxoflavin activities might be explained by its role as an active electron carrier between NADH and oxygen, involved in the production of reactive oxygen species such as hydrogen peroxides [67]. 

**Figure 2:**
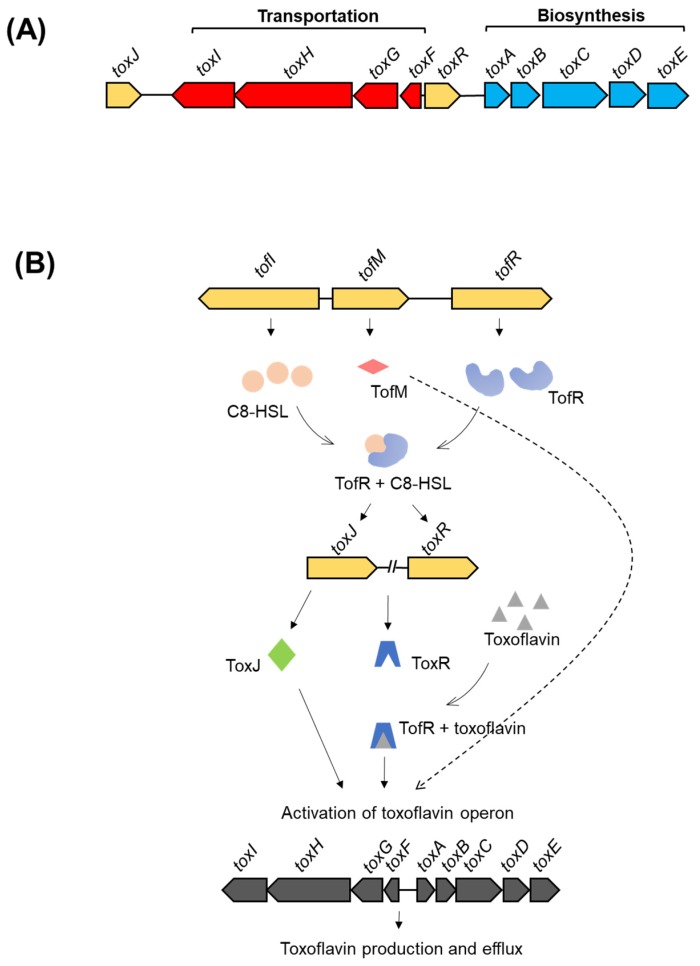
(**A**) A schematic representation of the *tox* operon for toxoflavin production and transport. Biosynthetic genes are shown in blue, transportation genes in red, and regulatory genes in yellow [69,70]. (**B**) An illustration of the proposed regulation of toxoflavin operons [70,71].

The genetic regulation of toxoflavin biosynthesis and transport is well characterized in *B. glumae*. A *tox* operon composed of five genes (*toxA*, *toxB*, *toxC*, *toxD*, and *toxE*) is responsible for toxin biosynthesis and is regulated by a LysR-type regulator, ToxR [68,69]. In a later study, another operon composed of four genes (*toxF*, *toxG*, *toxH*, and *toxI*) is responsible for toxoflavin transportation [70]. In the same study, the regulation of both *tox* operons is further elucidated. Upon activation by toxoflavin binding (as a co-inducer), ToxR regulates the expression of both operons. Moreover, the other regulator, ToxJ, is also involved in the activation of both *tox* operons. The activation of ToxJ is mediated by a quorum sensing (QS) system via the *luxI* homolog, *tofI*, and the *luxR* homolog, *tofR*, via AHL (*N*-acyl homoserine lactone) QS signals. That is, bacterial cell density is required for active toxoflavin production, so that bacterial cells can start toxoflavin-mediated attack of host cells only once a sufficiently high bacterial population has been reached [70]. An illustration of both *tox* operons and a proposed model of regulation is shown in Figure 2.

The complete genome sequence of *B. gladioli* BSR3 reveals the presence of the toxoflavin operons, as well as relevant regulatory genes [72]. To investigate the genetics of toxoflavin production and regulation in *B. gladioli*, Lee et al. [73] conducted a comparative genomic analysis of several strains of *B. gladioli*, including the non-toxoflavin-producing strain KACC11889 and *B. glumae* BGR1. The results indicate that the toxoflavin-producing strains of *B. gladioli* shared with *B. glumae* the genes for toxoflavin production and regulation, except that *toxJ* is located on another chromosome in *B. gladioli*. In addition, the genome of the non-toxoflavin-producing strain, *B. gladioli* KACC11889, contains the operons for biosynthesis and transport of toxoflavin, whereas the toxoflavin regulatory QS genes, *tofI* and *tofR*, are not found. Thus, the loss of these regulatory genes results in the inhibition of toxoflavin production, confirming the suggestion of a similar mechanism of toxoflavin regulation in *B. gladioli* and *B. glumae* [73].

Although *toxJ* regulatory genes are identified in the genome of the related species *B. plantarii*, toxoflavin biosynthesis and transport genes are not identified [29]. *B. plantarii* produces another phytotoxin, a non-benzenoid aromatic tropolone, which is responsible for seedling blight symptoms in rice, as confirmed by the replication of the characteristic bacterial-induced blight symptoms upon treatment of rice seedlings with exogenous tropolone [51,74]. Tropolone is also a potential iron chelator and has a wide range of biological roles such as antibacterial, antifungal, and insecticidal activities [75,76]. 

**Figure 3:**
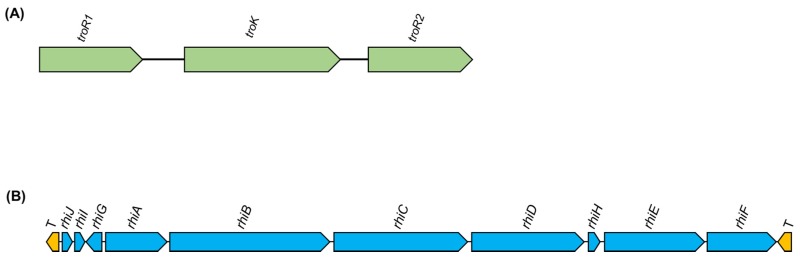
(**A**) A schematic representation of the two-component system genes (sensor histidine kinase, *troK* and the two-response regulator genes, *troR1* and *troR2*), which control the production of tropolone in *Burkholderia plantarii* (adapted from Miwa et al. [79]). (**B**) Schematic representation of the rhizoxin biosynthesis gene cluster (*rhi*). Rhizoxin biosynthetic genes are in blue and transposase genes in yellow. The annotation is adapted from Partida-Martinez et al. [25].

*B. plantarii* produces AHL-QS signal molecules resembling those of *B. glumae*, as the *luxI* family member, *plaI*, and the *luxR* family member, *plaR*, are highly similar to the *B. glumae* AHL-QS system genes, *tofI* and *tofR*. Through the generation of *plaR* knockout mutants, the role of the AHL-QS regulatory system in the virulence of *B. plantarii* to rice is established [77]. It was later revealed that treatment of *B. plantarii* with AHL-QS inhibitors resulted in the suppression of tropolone production by blocking the expression of *plaI* [78]. Therefore, a resemblance between the regulation of tropolone biosynthesis and that of toxoflavin, in *B. glumae*, could be inferred from the major role played by QS-related genes. This possibility is further supported by the presence of QS-AHL signal synthase-regulator loci within the tropolone biosynthesis operon (Seo et al., 2015 [29]). More recently, Miwa et al. [79] reported that a two-component signal transduction system regulates both tropolone biosynthesis and virulence in *B. plantarii*. Defects in any of the three genes encoding the two-component system (sensor histidine kinase and response regulators) resulted in the inhibition of toxin production and attenuation of bacterial virulence in rice. Three genes (i.e., *troR1*, *troK*, and *troR2*) are suggested to form an operon for tropolone synthesis, due to their adjacent chromosome position and their simultaneous requirement for toxin production [79]. A schematic organization of the two-component system genes controlling tropolone production is shown in Figure 3A. Further investigations are required to unravel the gene cluster organization and the regulation of tropolone biosynthesis, the main virulence factor in *B. plantarii*, and the possible regulatory roles of tropolone.

The other phytotoxin relevant for plant disease is rhizoxin, produced by *M. rhizoxinica*, the fungal endosymbiont of *R. microspores*. This bacterial endosymbiont alters the vegetative growth and sporulation of the host fungus by preventing the production of endosymbiont-free fungal spores [25,26]. The endosymbiont-produced rhizoxin is an effective inhibitor of eukaryotic cell growth by acting on *β*-tubulin and consequently blocking cell mitosis [80]. Besides its role as a virulence factor responsible for seedling blight symptoms in rice, various additional physiological functions have been attributed to rhizoxin, including reproduction in the host fungus and antibiotic activity against other microbes [25,81]. 

The availability and analysis of the complete genome sequence of *M. rhizoxinica* have allowed for the clarification of rhizoxin biosynthesis and the identification of the responsible gene cluster (*rhi*) [26,59]. The *rhi* locus is found on the large chromosome, flanked by transposase genes, indicating that it may represent a potentially mobile region of the genome [26,27]. It is also suggested that the rhizoxin gene cluster might have been transferred from the megaplasmid to the chromosome after horizontal acquisition, as concluded from the chromosomal location of the gene cluster, near the replication terminus, and the comparison of its G+C content with that of the rest of the chromosome [26]. Notably, in the comparative genomic analysis conducted by Seo et al. [29], genes involved in rhizoxin biosynthesis are also detected in other phytopathogenic *Burkholderia* spp., such as *B. plantarii* ATCC43733 and *B. glumae* PG1, for which the ability to produce rhizoxin has not been demonstrated. The organization on the *rhi* gene cluster is shown in Figure 3B.

The phytopathogen *R. andropogonis* produces the enol-ether amino acid phytotoxin, rhizobitoxine, that is responsible for leaf chlorosis in host plants [82]. Notably, rhizobitoxine has also been associated with a positive role in the symbiosis of legume nodulators. The phytotoxic activity of rhizobitoxine is related to inhibition of the methionine and ethylene biosynthetic pathways. The genes for rhizobitoxin biosynthesis (*rtx* operon) are identified in the legume nodulator, *Bradyrhizobium elkanii* [83,84]. However, further work is required to describe the biosynthetic genes and their regulation in *R. andropogonis*. 

Another example of the versatility of *Burkholderia* species and their contribution to vital ecological aspects is the phytotoxin produced by another *Burkholderia* endo-symbiont. In this case, the endo-symbiont is associated with the pine wood nematode, *Bursaphelenchus xylophilus*, causing pine wilt disease, a devastating threat to pine trees worldwide [85]. The pine wood nematode endo-symbiont *Burkholderia arboris* produces pyochelin, which is a bacterial siderophore with several bioactivities due to its capacity to generate reactive oxygen species [86]. However, *B. arboris*-produced pyochelin is responsible for damaging plant tissues and is linked to the development of pine wilt disease, as the treatment of pine callus with purified pyochelin confirms its strong phytotoxicity [87]. Further studies are required to unveil the phytotoxic mechanism of pyochelin.

The different phytotoxins produced by the phytopathogenic species of *Burkholderia* group, their effect, and mode of action are summarized in Table 2. 

#### 4.1.2. Secretion Systems

The ability of Gram-negative bacteria, including *Burkholderia* spp., to control the translocation of potent effectors from their sites of synthesis to the exterior of the cell, or even to other surrounding organisms, is a very important tool for communication and survival [88]. Six types of bacterial secretion systems have been discovered and differentiated based on their structure, function, or type of secreted effectors [89]. Species possessing more than one type of secretion system are also common [90].

Phytopathogenic *Burkholderia* spp. exhibit a wide spectrum of secretion systems, playing essential roles in adaptation and virulence. Kang et al. [91] confirmed the association of Type 2 secretion system (T2SS) and T3SS with functions related to host plant colonization and bacterial growth, as indicated by the reduced virulence and retarded growth of T2SS- and Hrp T3SS-deficient mutants in host plants. Comparative genomic analysis of phytopathogenic *Burkholderia* reveals that the genes for T3SS are highly conserved in several strains of *B. glumae*, *B. gladioli*, and *B. plantarii*. However, within species-variation is observed in T1SS and T4SS [29]. 

Phytopathogenic *Burkholderia* organisms have different groups of T6SSs. Group 1 is conserved in all species [92]. T6SS is involved in the delivery of effectors to both eukaryotic and prokaryotic cells. Thus, it is suggested to be important for the competition with other surrounding microbes and for virulence to host plants [93]. 

Although several functions have been reported for different bacterial secretion systems, for many reasons, including the remarkable diversity and limited expression of effectors as well as the multifarious nature of the secretion systems, not all secreted effectors have been identified and their exact mechanisms of action are still elusive [91,94,95]. 

#### 4.1.3. Other Virulence Factors

Several other factors are associated to the virulence of phytopathogenic *Burkholderia* species. Virulence-related enzymes such as lipases, proteases, and cellulases, as well as exopolysaccharides (EPS), contribute to the virulence of several species. For full virulence, the degradation of plant cell wall polymers by hydrolytic enzymes is crucial. Lipase is an important virulence factor for many bacterial pathogens, as it is responsible for the hydrolysis of the carboxyl ester bonds present in triacylglycerols, which liberate fatty acids and glycerol. Lipase could also play a role in the degradation of the epicuticle wax layer in plant tissues [96,97,98]. In *B. glumae*, lipase is also regulated by the AHL-QS system and is essential for panicle blight symptoms in rice [99]. The gene encoding lipase (LipA) is found in the three phytopathogenic species *B. glumae*, *B. gladioli*, and *B. plantarii* [29].

Pectin, mainly composed of polygalacturonic acid, is one of the major constituents of the plant cell wall. Polygalacturonases produced by phytopathogens can hydrolyze the pectic glycosidic bonds, depolymerizing pectin and causing rotting of plant tissues. *B. glumae* produces two different polygalacturonases (encoded by *peh*A and *peh*B) that are possibly regulated by QS and secreted by a T2SS. The *pehA* locus is present in *B. glumae* but not detected in *B. gladioli* and *B. plantarii* [29]. 

The EPSs have been shown to contribute to the pathogenesis of several plant pathogens by causing occlusion in the host vascular systems, leading to plant wilting [100]. Although the role of EPSs in the epidemiology of rice seedling blight has not yet been clearly established, they could explain the wide range of crops showing wilting symptoms in response to *B. glumae* infection [46]. In *T. caryophylli*, EPSs are suggested as important virulence factors, responsible for bacteria–plant interactions [101]. Further investigations are required to elucidate the contribution of EPSs to the virulence of phytopathogenic *Burkholderia*. 

## 5. Plant-Beneficial and Symbiotic *Burkholderia* Species

If, on the one hand, pathogenic *Burkholderia* species are the subject of intensive research, mainly because of their economic implications, on the other hand, plant-beneficial and symbiotic species have attracted the attention of scientists due to their impressive ability to promote plant physiology and growth. The main intriguing findings about this group of bacteria are the discovery of nitrogen fixation ability [102] and the identification of plant endosymbionts and legume nodulators [103,104]. As a result of multiple taxonomic rearrangements and updates (see Section 2), the majority of plant-beneficial and symbiotic strains increasingly clustered together in separate clades, mainly within the *Paraburkholderia* group, based on the presence of several common features such as nitrogen fixation, metabolic versatility, and plant growth promoting ability [105]. Comparative genomic analyses between beneficial plant-associated *Burkholderia* species revealed a high degree of genetic synteny in their large multireplicon genomes [28]. Previous studies focusing on genomics and metabolism have reported the capacity of this *Burkholderia* subgroup to support healthy plant growth.

### 5.1. Benevolence Factors in Plant-Beneficial and Symbiotic Burkholderia

Beneficial plant-associated species of the *Burkholderia* sensu lato group have been reported to carry out several functions that are beneficial to plants, such as colonization of plant surfaces and rhizosphere, nitrogen fixation, production of siderophores, solubilization of phosphates, metabolism of volatile compounds, degradation of pollutants, production of plant growth-promoting phytohormones, reduction of plant ethylene levels, and protection from biotic and abiotic stress [106,107,108].

#### 5.1.1. Colonization of Plant Tissues and the Role of EPSs

The ability of plant-beneficial bacteria to colonize the surface of vegetal tissues and the rhizosphere is important for the promotion of healthy plant growth. In this respect, the production of EPSs is essential not only to enhance bacterial tolerance to environmental stress but also for efficient colonization, by adhesion to the root surface, and for the aggregation of root-adhering soil particles [100,109]. Bacterial EPSs also play a key role in the interaction between the plant and the endophytic or associated bacteria. It has been suggested that bacteria–plant symbiosis in the *β*-rhizobia is initiated by the bacterial production of an infection thread allowing bacteria to invade and colonize plant roots. Jones et al. [110] reported that, unlike wild-type bacteria, mutants deficient in EPS production are unable to initiate a symbiotic relationship with a host plant. EPS-producing bacteria regulate the expression of plant defense-related genes to prepare plants for colonization. These findings suggest that bacterial EPSs could serve as signals regulating gene expression in plants [110]. Several members of plant-beneficial *Burkholderia*, such as *Paraburkholderia tropica*, *Paraburkholderia caribensis*, *Paraburkholderia phytofirmans*, and *Paraburkholderia kururiensis*, have been reported to synthesize EPSs, the structure of which has been elucidated [111,112,113,114].

#### 5.1.2. Nitrogen Fixation in Diazotrophic and Legume Nodulator *Burkholderia*

In *Burkholderia* bacteria, biological nitrogen fixation has been found in both plant endosymbiotic diazotrophic non-legume nodulator species, such as *P. phytofirmans* and *Paraburkholderia unamae*, and in legume nodulator species such as *Paraburkholderia tuberum* and *P. phymatum* [102,115]. 

The nitrogenase enzyme responsible for nitrogen fixation is composed of two structurally conserved components. Component I is encoded by *nifD* and *nifK* and component II is encoded mainly by *nifH*, forming the basic regions of the *nif* operon [116,117,118]. The *nifHDK* operon was first reported in the *Burkholderia* group in an endosymbiotic species of the arbuscular mycorrhizal fungus, *Gigaspora margarita* [118]. The presence of *nifHDK* coupled with nitrogen fixation has been confirmed in several other species in the *Burkholderia* group [102]. Moreover, the *fix* genes are also essential for nitrogen fixation, especially in microaerobic conditions. The *nifHDK* is controlled by the transcriptional regulator NifA. *NifA* and *nifHDK*, along with the *fix* genes, are regulated through the two-component signal transduction system through FixL and FixJ [119,120]. 

Legume nodulation is defined as the induction of nodule formation in plants by certain bacteria species, which fix atmospheric nitrogen for plants and receive carbons in exchange [121]. Previously, legume nodulation was thought to be an exclusive feature of the Rhizobiaceae members of *α*-Proteobacteria [122]. However, with the identification of *Burkholderia* legume nodulators, this hypothesis was disproved, and the term *β*-rhizobia was proposed to describe the legume nodulators in the *β*-proteobacteria [103,123,124,125,126,127]. Since then, several other *Burkholderia* species have been reported as legume nodulators [15]. 

The nodulation genes (*nod*), encoding proteins for production, transport, and regulation of the nod factor, along with the *nif* genes for nitrogen fixation, are considered as genes essential for the *β*-rhizobia symbiosis and are reported to be directly involved in *Burkholderia* nodulation ability [11]. The phylogenetic analyses conducted on the symbiosis-related genes, *nodC* and *nifH*, have evidenced that symbiosis is an ancient function in *Burkholderia* and these genes are vertically inherited and not the result of recent horizontal acquisition [104]. In contrast, in a recent study by Estrada-de los Santos et al. [22], phylogenies constructed by using *nifD* and *nifH* for nitrogen fixation and *nodABCD* for nodulation provided evidence that the *nif* genes have been acquired via horizontal gene transfer [22]. In the same study, the *nif* genes are widely present among *Burkholderia* sensu lato bacteria, especially in the *Paraburkholderia* group of plant-associated legume nodulators. However, *nif* genes are not found in *Caballeronia*, *Robbsia* or the newly assigned genus of fungal endosymbionts, *Mycetohabitans*. 

**Figure 4:**
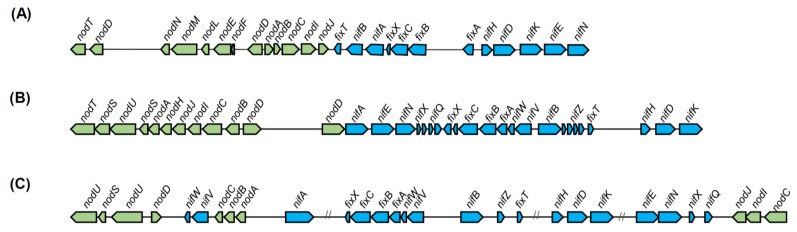
Schematic illustration of *nod* and *nif/fix* gene cluster organization in representative species: (**A**) α-rhizobia *Rhizobium leguminosarum*; (**B**) the mimosoid-nodulator, *Paraburkholderia phymatum*; and (**C**) the papilionoid-nodulator, *Paraburkholderia tuberum*. *Nod* genes are shown in green and *nif/fix* genes are in blue. The “//” symbol means an interruption by other genes, some of which are symbiosis-related. The annotation is adapted from De Meyer et al. [120].

With regard to the *nod* genes, which are only found in the group of nodulators, phylogenetic analysis has divided the nodulators into two clades based on the respective host plants: the mimosoid nodulator and the papilionoids nodulator clades. The divergence in the *nod* genes among the *Paraburkholderia* nodulators seems to be related to host plant specificity [120]. A more in-depth evaluation of *nod* gene organization supports the distinction between mimosoid and papilionoids nodulators. In the mimosoid-nodulators, the *nod* operon was organized as a single copy of *nodBCIJHASU*, differing from the related *α*-rhizobia and the papilionoid-nodulators [120]. The genetic organization of the *nod* and *nif/fix* gene clusters of representative species of *α*-rhizobia, mimosoid and papilionoid *Paraburkholderia* nodulators is shown in Figure 4. The papilionoid nodulators’ *nod* genes could have been acquired from the related *α*-rhizobia, whereas a different evolutionary origin is suggested for the *nod* genes of mimosoid nodulators [22].

#### 5.1.3. Plant Growth Promotion

Plant growth promotion is common among members of the beneficial plant-associated *Burkholderia*, which possess several growth promoting factors involved not only in nitrogen fixation but also in ACC (1-aminocyclopropane-1-carboxylate) deaminase activity, phosphate solubilization, as well as siderophore and phytohormone production [105]. 

In plant tissues, ACC is an intermediate precursor in the production of the plant growth inhibitor, ethylene, in developing or stressed plants. The bacterially produced ACC deaminase enzyme catalyzes the conversion of ACC to ammonia and *α*-ketobutyrate, thereby reducing the ethylene levels in plant tissues, which in turn results in plant growth promotion, mainly manifesting as root elongation [128]. Production of ACC deaminase is a common feature in beneficial plant-associated *Burkholderia* species and the relevant encoding gene (*acdS*) is highly conserved and widely distributed among the rhizospheric, endophytic, and nodulator groups [129]. Furthermore, ACC deaminase activity is confirmed by comparing tomato plant responses to inoculation with an ACC deaminase-producing wild-type *P. unamae* strain and an *acdS* knockout mutant, which clearly demonstrates the plant growth-promoting properties of ACC deaminase-producing *P. unamae* [129]. In another study, an *asdS* deletion mutant of *P. phytofirmans*, devoid of ACC deaminase activity, lacked the ability to promote the root elongation of canola seedlings [130]. 

Phosphorus is an essential macronutrient for plant growth. Soil mineral and organic phosphates can be solubilized and become available for plants by the action of beneficial soil microbes [131]. A phosphate solubilization ability is reported in several species of plant-associated *Paraburkholderia* [132,133,134]. Among the species tested by Estrada-de los Santos et al. [22], only the soil bacterium *Trinickia dabaoshanensis* (formerly *Paraburkholderia dabaoshanensis*) can solubilize phosphate. The genetic background and mechanisms of phosphate solubilization have not been clearly characterized yet.

Plant growth regulators such as the auxin class of phytohormones are known to promote plant growth, particularly by controlling growth and development of plant roots [135]. Indole-3-acetic acid (IAA) is the most common and naturally occurring auxin and its presence has been extensively documented in several soil and rhizosphere bacteria. A clear example of IAA-producing species, among beneficial plant-associated *Burkholderia*, is the well-known plant growth-promoting *P. phytofirmans*, in which whole-genome sequence analysis reveals the presence of two independent putative IAA synthesis pathways, the indole-3-acetamide and the tryptophan side-chain oxidase pathway [136,137]. More recently, Zúñiga et al. [138], reported that IAA also plays a role in plant–microbe interactions (cell–cell communication) and that *P. phytofirmans* is not only a producer of IAA but can also degrade excess exogenous IAA that could suppress plant growth. The production of IAA by *P. phytofirmans* and its association with plant growth promotion and colonization were further confirmed by Naveed et al. [139] on maize plant, who found it to be dependent on L-Tryptophan. 

#### 5.1.4. Other Benevolence Factors in Plant-Associated *Burkholderia*

In addition to the above described beneficial effects of plant-associated *Burkholderia*, other plant-beneficial roles have been described. Several species exert antagonistic activities against phytopathogens including fungi, bacteria, and even nematodes. Broad-spectrum antibiotic activity is reported in a strain of *Burkholderia ambifaria*, exhibiting an effective control over several soil fungal pathogens [140]. Antifungal activity is reported in several other species, e.g., *Paraburkholderia bryophila*, *Paraburkholderia megapolitana*, *Paraburkholderia ginsengiterrae*, and *Paraburkholderia panaciterrae* [141,142]. Along with several other beneficial plant-associated *Burkholderia*, the latter species have also been found to produce siderophores, allowing them to compete for the available iron and dominate over pathogenic microbes [106]. In addition, the induction of resistance against biotic and abiotic stress was also reported, mainly by *P. phytofirmans*, as bacterial treatment triggers resistance against gray mold in grapevine and to improve plant tolerance to cold stress [143,144].

Another type of *Burkholderia*-beneficial interactions with plants is the intimate association of the plant leaf-nodulating *Candidatus* Burkholderia. Although the attempts to cultivate this group of bacteria remain unsuccessful, culture-independent molecular analyses are helpful to reveal the taxonomic position of these bacteria within the *Burkholderia* assemblage and the importance of this association to host plant [145]. The association of Ca. Burkholderia with leaves of *Ardisia*, *Pavetta*, *Psychotria*, and *Serricanthe* genera is vertically transmitted (i.e., transmitted from one generation to the next through seeds by incorporation to the reproductive stages), very specific and critical for the host plant development as the heat-treated seeds that germinate into aposymbiotic seedlings cannot reach maturity [146,147]. The genomic and functional studies on *Candidatus* Burkholderia kirkii and *Candidatus* Burkholderia crenata provide insights into secondary metabolism and possible beneficial roles to host plant such as the biosynthesis of antibiotic and insecticidal compounds for protection [145,148,149]. Future work is required to further characterize the mechanisms and roles of the unique symbiotic leaf–nodulating Ca. Burkholderia, which differs from the known symbiosis.

The main plant-associated phytopathogenic and beneficial species of the *Burkholderia* group, as well as their major virulence or benevolence factors, are summarized in Table 3. 

## 6. Conclusions and Future Perspectives

*Burkholderia* organisms are ubiquitous in diverse environmental niches and display a high adaptability to changing environments. Unique genomic features and a large encoding capacity provide this group of bacteria with the flexibility required for adaptation to various lifestyles. Recent advancements in sequencing techniques and bioinformatics tools have led to a remarkable increase in the availability of genome sequences, improving our comprehension of the relationships between different *Burkholderia* species, their taxonomy, the mechanisms underlying their versatility and the potential factors involved in bacterial virulence or benevolence. The phytopathogenic *Burkholderia* group encompasses several important etiological agents of plant disease, mainly causing significant loss of rice crops. Comparative genomic analyses combined with functional and phenotypic studies have generated new knowledge of disease-causing mechanisms and involved virulence factors. However, further research efforts are needed to better characterize the virulence factors of phytopathogenic *Burkholderia*, their genetic background, and regulation.

Regarding the beneficial plant-associated *Burkholderia*, although several studies have reported efficient plant growth-promoting and potential biocontrol activities of several members of *Burkholderia* spp., their use in agriculture has been limited due to concerns related to their similarity with opportunistic human pathogens. However, based on current knowledge, including refined taxonomy, as well as the absence of pathogenic hallmarks in their genomes, a clear distinction between these bacteria and human pathogens has been established, suggesting that, in the coming years, members of the beneficial *Burkholderia* spp. could be utilized as suitable alternatives to agrochemicals to promote plant growth, manage plant diseases, and for biotechnological applications

## Figures and Tables

**Table 1:** An overview of the major rearrangements and updates in the taxonomy of *Burkholderia*.

Year	Finding	Details	Reference
1942	First isolation of *Burkholderia*	Originally named *Phytomonas caryophylli*; then *Pseudomonas caryophylli*	[5]
1992	A new *Burkholderia* genus was proposed	The new genus comprised seven species from the genus *Pseudomonas*	[8]
2011	A second genus (*Caballeronia*) was suggested	Based on phylogenetic analysis of multiple genes and comparative genomics; however, the evidence was not sufficient to confirm the new grouping	[15]
2014	The genus *Paraburkholderia* was proposed	Based on the analysis of conserved sequence in/dels	[18]
2016	Inclusion of several species in the *Paraburkholderia* genus and establishment of the *Caballeronia* genus	Eleven species were reclassified as *Paraburkholderia* and 14 species were transferred to the newly established *Caballeronia* genus	[19]
2017	*Burkholderia andropogonis* was separated in a newly proposed genus as *Robbsia andropogonis*	Based on multilocus sequence, 16S rRNA gene phylogeny, and average nucleotide identity analyses, as well as tetranucleotide signature frequency and percentage of conserved proteins	[20]
2017	Confirmation of the genetic boundaries among the 4 established groups and suggestion of a fifth division: *Paraburkholderia rhizoxinica*	Five groups (*Burkholderia* sensu stricto, *Paraburkholderia, Caballeronia, Robbsia, Paraburkholderia rhizoxinica*) were separated based on maximum likelihood phylogenies using the amino acid and nucleotide sequence of 106 conserved proteins	[21]
2018	Two novel genera (*Mycetohabitans* and *Trinickia*) were proposed	Based on whole-genome comparative study and phylogenetic analysis of conserved genes	[22]

**Table 2:** Phytotoxins produced by phytopathogenic *Burkholderia* species, their major phytotoxic effect, and mode of action.

Phytotoxin	Producing Species	Major Phytotoxic Effect	Mode of Action	References
Toxoflavin	*B. glumae*; *B. gladioli*	Severe damage to rice panicles and inhibition of sprout and root elongation in seedlings.	An active electron carrier between NADH and oxygen, producing reactive oxygen species	[65,67]
Tropolone	*B. plantarii*	Blight symptoms	A potential iron chelator with multiple biological roles	[51,76]
Rhizoxin	Mycetohabitans rhizoxinia *	Seedling blight symptoms in rice; signaling element for bacterial-fungal symbiosis	Acts on β-tubulin and blocks mitosis, inhibiting eukaryotic cell growth.	[25,80,81]
Rhizobitoxine	*Robbsia andropogonis*	Leaf chlorosis in host plants	Inhibition of methionine and ethylene biosynthetic pathways	[82,84]
Pyochelin	*Burkholderia arboris*	Necrosis in *Pinus densiflora*	ND	[87]

* M. rhizoxinica is considered as a phytopathogenic species, as it is responsible for the production of rhizoxin, the major virulence factor for rice seedling blight.

**Table 3:** A list of the main Phytopathogenic and plant-associated beneficial *Burkholderia* species, their major virulence or benevolence factor, and host or isolation sources.

Category	Species	Host/Isolation	Major Virulence/Benevolence Factors	Reference
**Phytopathogenic species**				
	*Burkholderia gladioli*	Gladiolus, Onion, Rice	Toxoflavin, Lipase, T3SS	[47,48,49,50]
	*Burkholderia glumae*	Rice and several other crops	Toxoflavin, Lipase, T3SS, T6SS, EPSs, polygalacturonases	[43,44]
	*Burkholderia plantarii*	Rice and several other crops	Tropolone, Lipase, T3SS	[42,51]
	*Trinickia caryophylli*	Carnation and onion	EPSs	[22,56,57,101]
	*Mycetohabitans rhizoxinia*	In combination with host fungus causing rice seedling rot	Rhizoxin, T3SS	[22,42,59]
	*Robbsia andropogonis*	Sorghum, velvet beans, orchids, carnation	Rhizobitoxine	[42,53,54,55,82]
**Plant beneficial species**				
Free-living and endophytic				
	*Paraburkholderia phytofirmans*	Cereal and other crop soils	nif, ACC deaminase, EPSs, IAA	[113,129,136,137]
	*Paraburkholderia xenovorans*	Rhizosphere	nif, ACC deaminase	[106,129]
	*Paraburkholderia unamae*	Corn, sugarcane, coffee plants, and rhizosphere	nif, ACC deaminase	[106,129]
	*Paraburkholderia silvatlantica*	Corn rhizosphere	nif, ACC deaminase	[107,129]
	*Paraburkholderia graminis*	Rhizosphere	ACC deaminase	[129]
	*Paraburkholderia bryophila*	Moss gametophytes	Siderophore, antifungal activity, phosphate solubilization	[106,134,141]
	*Paraburkholderia kururiensis*	Aquifer sample	nif, ACC deaminase, EPSs	[102,114,129]
	*Paraburkholderia ginsengiterrae*	Ginseng rhizosphere	Antifungal activity	[142]
	*Paraburkholderia panaciterrae*	Ginseng rhizosphere	Antifungal activity	[142]
	*Caballeronia glathei*	Fossil lateritic soil	nif	[150]
	*Paraburkholderia heleia*	aquatic plant from highly acidic swamps	nif	[151]
	*Paraburkholderia megapolitana*	Moss gametophytes	Siderophore, antifungal activity	[106,141]
	*Paraburkholderia terrae*	Forest soil	nif	[152]
	*Paraburkholderia tropica*	Sugarcane	nif, EPSs, phosphate solubilization	[106,112,133,153]
Legume nodulators				
	*Paraburkholderia phenoliruptrix*	Mimosa root nodules	nod, nif, ACC deaminase	[129,154]
	*Paraburkholderia phymatum*	Root nodules of mimosa and other tropical legumes	nod, nif, ACC deaminase	[123,127,129,155]
	*Paraburkholderia tuberum*	Root nodules of Papilionoid and tropical legumes	nod, nif, ACC deaminase	[123,129]
	*Paraburkholderia mimosarum*	Mimosa root nodules	nod, nif	[126]
	*Paraburkholderia nodosa*	Mimosa root nodules	nod, nif, biocontrol activity	[156,157]
	*Paraburkholderia caballeronis*	Rhizosphere of tomato	nod, nif	[158]
	*Paraburkholderia caribensis*	vertisol	nod, ACC deaminase, EPSs	[111,124,129,159]
	*Paraburkholderia diazotrophica*	Mimosa nodules	nod, nif	[160]
	*Paraburkholderia dilworthii*	*Lebeckia ambigua* root nodules	nod, nif	[161]
	*Paraburkholderia dipogonis*	Papilionoid legume nodules	nod, nif	[162]
	*Paraburkholderia kirstenboschensis*	Papilionoid legume nodules	nod, nif	[163]
	*Paraburkholderia rhynchosiae*	*Rhynchosia ferulifolia* legume	nod, nif	[164]
	*Paraburkholderia sabiae*	Mimosa nodules	nod, nif	[165]
	*Trinickia symbiotica*	Mimosa nodules	nod, nif, siderophore	[22,166]

Member that have been isolated from human clinical samples are excluded from the list of plant-associated beneficial species. Nod, can form nodules in legumes; nif, nitrogen fixation; EPSs, exopolysaccharides; T3SS, Type 3 secretion system; T6SS, Type 6 secretion system; IAA, indole acetic acid.

## Abbreviations

BCC	*Burkholderia cepacia* complex
Mbp	Million base pair
QS	Quorum sensing
AHL	Acyl homoserine lactone
TSS	Type secretion systems
EPS	Exopolysaccharide
ACC	Aminocyclopropane-1-carboxylate
IAA	Indole-3-acetic acid
